# Hepatitis E Virus (HEV) Seroprevalence in the general population of the Republic of Korea in 2007–2009: a nationwide cross-sectional study

**DOI:** 10.1186/1471-2334-14-517

**Published:** 2014-09-24

**Authors:** Youngsil Yoon, Hye Sook Jeong, Haesun Yun, Hyeokjin Lee, Yoo-Sung Hwang, Bohyun Park, Chae Jin Lee, Sangwon Lee, Ji-Yeon Hyeon

**Affiliations:** Division of Vaccine Research, Korea National Institute of Health, Korea Centers for Disease Control and Prevention, Osong-eup, CheongJu, Chungcheongbuk-do 363-951 Republic of Korea; Neodin Medical Institute, Seongdong-gu, Seoul 133-170 Republic of Korea; Division of Infectious Diseases Control, Korea Centers for Disease Control and Prevention, Osong-eup, CheongJu, Chungcheongbuk-do 363-951 Republic of Korea; Division of Biosafety Evaluation and Control, Korea National Institute of Health, Korea Centers for Disease Control and Prevention, Osong-eup, CheongJu, Chungcheongbuk-do 363-951 Republic of Korea; Division of General Affairs, Korea Centers for Disease Control and Prevention, Osong-eup, CheongJu, Chungcheongbuk-do 363-951 Republic of Korea

**Keywords:** Hepatitis E virus, Seroprevalence, National survey, Republic of Korea

## Abstract

**Background:**

Hepatitis E virus (HEV) is an emerging pathogen associated with endemic and acute viral hepatitis. In this study, we investigate the HEV seroprevalence and putative risk factors by a nationwide cross-sectional study in the Republic of Korea.

**Methods:**

The prevalence of anti-HEV antibody was investigated in 2,450 serum samples collected in fourth Korea National Health and Nutrition Examination Survey. In addition, epidemiological information on possible risk factors including gender, age, education, occupation, and residence location for exposure to HEV was obtained.

**Results:**

The frequency of anti-EIA reactive sample was 5.9% (144/2450). The individuals in groups with male, older age, low education level and living in rural or coastal regions had high seroprevalence estimates (*P* ≤ 0.001). In addition, seroprevalence was significantly higher among individuals with self-identified skilled agricultural, forestry, and fishery workers (31.3%, *P* < 0.001).

**Conclusions:**

This study provides valuable data that could be used to investigate associations of HEV seroprevalence and putative risk factors by a nationwide cross-sectional study. The high HEV seroprevalence of skilled agricultural, forestry, and fishery workers and individuals lived in coastal and rural area indicated that zoonotic transmission is an important risk factor for HEV infection in the republic of Korea. Further studies that include detailed and continuous nationwide surveys are required to identify unrecognized risk factors and to monitor the HEV infection prevalence.

**Electronic supplementary material:**

The online version of this article (doi:10.1186/1471-2334-14-517) contains supplementary material, which is available to authorized users.

## Background

Hepatitis E virus (HEV) is a member of the genus *Hepevirus*, family *Hepeviridae*, and is a non-enveloped virus with a single-stranded, positive-sense RNA genome of approximately 7.5 kb [1]. It usually causes mild disease, inflammation of the liver, and the overall mortality rate of HEV-infected individuals is approximately 1% [2]. However, the mortality rate is significantly higher (20 ~ 30%) among HEV-infected pregnant women in endemic countries such as India and Pakistan [3, 4]. The epidemiology of HEV infection can be divided into two patterns [5]. The first is an outbreak pattern in areas of high endemicity such as Central and Southeast Asia, Middle East, North Africa, and India [6, 7]. This pattern usually has been associated with fecal-to-oral transmission of HEV genotype 1 and 2 [4, 8]. HEV infection in industrialized countries is generally associated with travel to these endemic areas. The second is a sporadic pattern that is seen in worldwide and occurs via zoonotic transmission and foodborne transmission such as consumption of raw meat obtained from infected swine, deer, or boar [9–11]. In previous studies, anti-HEV antibodies were detected in pigs, cattle, rodents, and monkeys, and zoonotic transmission from pigs has been reported [7, 12]. In addition, HEV genotype 3 and 4 are detected in swine, wild boar, deer, and rabbit and is associated with autochthonous infection in industrialized countries [4, 13] In previous studies, the overall prevalence of anti-HEV antibodies in human and animals were about 20% in human, 31.5% in dogs, 14.8% in swine, 10.1% in cats, and 3.5% in cattle in the Republic of Korea [14–16]. In addition, HEV genotype 3 or genotype 4 were isolated from human, swine, wild boar, and oyster in the Republic of Korea [12, 14, 15, 17]. Although there is an increased research interest in HEV, the exact routes of transmission remain unclear and are merely based on case reports. The several cases by zoonotic (foodborne) transmission and blood transfusion were reported, but these are unlikely to explain all HEV cases [7, 9]. Thus, the study about the transmission route and risk profile of HEV infection would be needed to better understand the epidemiological profile of HEV.

The aim of this study was to investigate associations of HEV seroprevalence and putative risk factors by a nationwide cross-sectional study.

## Methods

### Study population

A total of 2,450 individuals were randomly selected from 7,576 participants of the fourth Korea National Health and Nutrition Examination Survey (KNHANES IV) that was conducted between 2007 and 2009.

KNHANES is nationally representative cross-sectional survey and has been conducted by the Korea Centers for Disease Control and Prevention (KCDC) based on the National Health Promotion Act [18]. It used a rolling sampling survey design that involved a stratified, multi-stage, clustered probability sampling. The details about KNHANES IV were reported in Kweon *et al*’s study [18].

The participants were grouped on the basis of their age into 10-year age groupings and the age of the survey participants ranged between 10–55 years. Residence with a population of >200,000 was defined as urban area, and all others were defined as rural area. Region was categorized to coastal and inland area by Coastal Management Act of Korea Ministry of Land, Transport, and Maritime affairs. The individuals of coastal area group resided within 1 km from coastline, and the others not defined as coastal area was defined as inland area group. The anti-HEV IgG analysis was performed with the selected sample of 2,450 individuals on the basis of their residential area in each age stratum.

Serum samples were stored at -80°C until required for analysis at the National Biobank of Korea (Seoul, Korea). Informed consent was provided by all of the participants in the KNHANES IV study, and the study was approved by the Institutional Review Board of the Neodin Medical Institute (Seoul) prior to initiation.

### Enzyme-linked immunosorbent assay (ELISA) for anti-HEV IgG

We estimated the presence of anti-HEV immunoglobulin G (IgG) by using a commercial HEV IgG ELISA kit according to the manufacturer’s instructions (Beijing Wantai Biological Pharmacy Enterprise, Beijing, China), and the results were presented as frequency of anti-HEV reactivity. This kit was produced with a recombinant peptide corresponding to amino-acid residues 396 to 606 of the major structural protein of the HEV [19]. Serum samples with absorbance values greater than or equal to the cut-off value (0.16 in this study) were considered positive. ELISA tests were performed duplicate in all samples.

### Statistical analysis

The participant demographic and socioeconomic status including gender, age, educational achievements, occupation, and region were derived from their study questionnaire. The association of frequency of anti-HEV EIA reactivity and environmental risk factors was assessed by using logistic regression and chi-squared tests. Differences were considered to be statistically significant at *P* < 0.05. Data analyses were performed by using the statistical package SAS (version 9.1, SAS Institute, Cary, NC, USA).

### Ethical approval

This study was approved by the Institutional Review Board of the Neodin Medical Institute (2011–01).

## Results

The overall frequency of anti-HEV IgG EIA reactivity was found to be 5.9% (144/2,450). Furthermore, 7.8% (77/994) male participants and 4.6% (67/1,456) female participants were seropositive (*P* = 0.0001) (Table 1). Data analysis using age-group stratification indicated a significant increase in frequency of anti-HEV IgG EIA reactivity with increasing age (*P* < 0.0001). The frequency of anti-HEV IgG EIA reactivity of individuals in their 20s 30s, 40s, and 50s were 1.2% (9/521), 2.4% (13/553), 12.0% (67/557), and 20.9% (57/273), respectively with increasing odd ratios (ORs) from 6.3 to 143.8 (Table 1).

**Table 1:** **Frequency of anti-HEV IgG EIA reactivity associated with gender and age among participants in the Korea National Health and Nutrition Examination Survey, 2007–2009**

Variable	Tested (n = 2,450)	Positive for anti-HEV, N (%)	*P*value*	OR (95% CI)
Overall prevalence	2,450	144 (5.9)	-	
Sex				
Male	994	77 (7.8)	0.001	1.0
Female	1,456	67 (4.6)		0.6 (0.4-0.8)
Age (years)				
10–19	546	1 (0.2)	<.0001	1.0
20–29	521	6 (1.2)		6.3 (0.8-52.9)
30–39	553	13 (2.4)		13.1 (1.7-100.6)
40–49	557	67 (12.0)		74.5 (10.3-538.5)
50–55	273	57 (20.9)		143.8 (19.8- >999.9)

*If *P* < 0.05, the differences of seroprevalence among compared groups are statistically significant.

To better understand the epidemiological profile and risk factor of HEV infection, the association of seropositive rates and educational level, occupation, and residence location were assessed. Groups with educational levels of elementary school or below, middle school graduate, high school graduate and university graduate had seropositive individual rates of 20.5% (27/132), 15.1% (23/152), 9.0% (56/621) and 6.6% (31/473), respectively (*P* < 0.0001) (Table 2).

**Table 2:** **Frequency of anti-HEV IgG EIA reactivity associated with educational level, occupation and region of the participants in the Korea National Health and Nutrition Examination Survey, 2007–2009**

Variable	Tested^a^	Positive for anti-HEV, N (%)	*P*value^b^	OR (95% CI)
Educational level^c^				
Elementary school or below	132	27 (20.5)	<.0001	1.0
Middle school graduate	152	23 (15.1)		0.7 (0.4-1.3)
High school graduate	621	56 (9.0)		0.4 (0.2-0.6)
University graduate	473	31 (6.6)		0.3 (0.2-0.5)
Occupation				
Managers, Professionals	295	19 (6.4)	<.0001	1.0
Clerks	234	14 (6.0)		0.9 (0.5-1.9)
Service provider and Seller	357	26 (7.3)		1.1 (0.6-2.1)
Skilled Agricultural, Forestry and Fishery Workers	48	15 (31.3)		6.6 (3.1-14.2)
Equipment, Machine Operators and Manufacturing Workers	230	20 (8.7)		1.4 (0.7-2.7)
Elementary Workers	151	20 (13.3)		2.2 (1.1-4.3)
Unemployed (housewife, student, etc.)	819	29 (3.5)		0.5 (0.3-0.9)
Residence				
Urban area	1,937	90 (4.7)	<.0001	1.0
Rural area	513	54 (10.5)		0.4 (0.3-0.6)
Region of residence				
Coastal area	1,064	90 (8.5)	<.0001	1.0
Inland area	1,386	54 (3.9)		2.4 (1.7-3.4)

^a^The total no. of participants shown is <2,450, owing to deficient reporting.

^b^If *P* < 0.05, the differences of seroprevalence among compared groups are statistically significant.

^c^The seovprevalence of individuals over the age of 10 years old were used for this data.

The skilled agricultural, forestry, and fishery workers showed the highest frequency of anti-HEV IgG EIA reactivity (31.3%, 15/48) and associated with significantly high odd of HEV seropositivity (OR, 6.6 [95% CI, 3.1-14.2]) (Table 2). The seropositive rates for elementary workers also showed high frequency of anti-HEV IgG EIA reactivity (13.3%, 20/151) (OR, 2.2 [95% CI, 1.1-4.3]). However, the frequency of anti-HEV IgG EIA reactivity in equipment, machine operators and manufacturing workers (8.7%, 20/230), service provider and seller (7.3%, 26/357), managers and professionals (6.4%, 19/295), and clerks (6.0%, 14/234) were lower than other occupation groups (Table 2).

**Figure 1:**
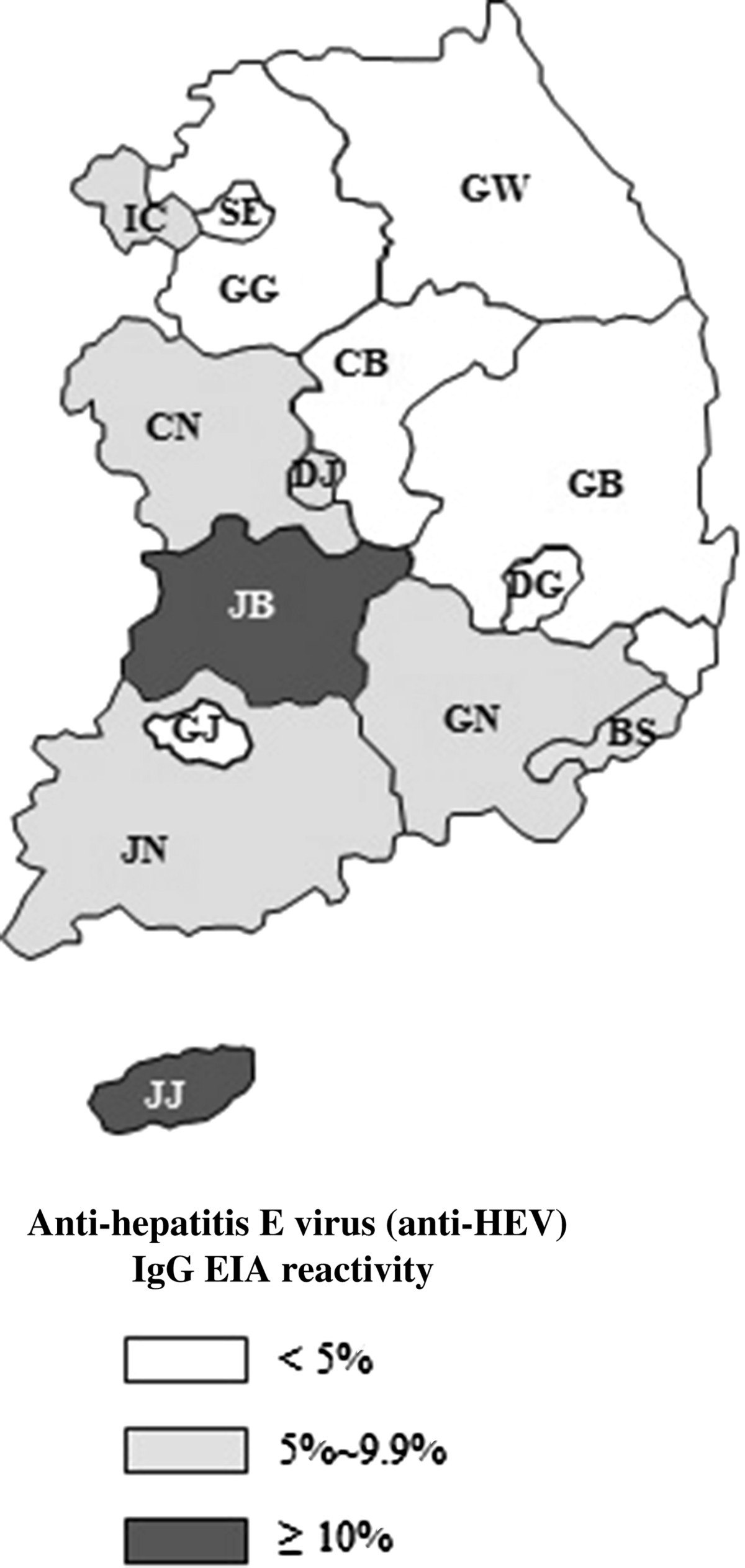
**Map of Korea showing a frequency of anti-hepatitis E virus (anti-HEV) Ig G EIA reactivity in the 16 regions of the Korea National Health and Nutrition Examination Survey, 2007–2009.** GW, Gangwon-do; GG, Gyeonggi-do; GN, Gyeongsangnam-do; GB, Gyeongsangbuk-do; GJ, Gwangju; DG, Daegu; DJ, Daejeon; BS, Busan; SE, Seoul; US, Ulsan; IC, Incheon; JN, Jeollanam-do; JB, Jeollabuk-do; JJ, Jeju-do; CN, Chungchengnam-do; CB, Chungchengbuk-do.

The incidence of HEV seropositive individuals was the highest in Jeju-do (15.0%, 16/107), followed by Jeollabuk-do (12.5%, 11/88), Jeollanam-do (9.4%, 20/212), and Chungcheongnam-do (9.3%, 11/118; Figure 1). The coastal/inland, urban/rural residence classification showed differences in frequency of anti-HEV IgG EIA reactivity based on the area of residence. The coastal areas demonstrated a significantly higher frequency of anti-HEV IgG EIA reactivity (8.5%, 90/1,064) than the inland areas (3.9%, 54/1,386; Table 2). The rural areas had a significantly higher frequency of anti-HEV IgG EIA reactivity (10.5%, 54/513) than that of the urban areas (4.7%, 90/1,937; Table 2).

## Discussion and conclusions

On the basis of the testing of 2,450 KNHANES IV study participants, the overall frequency of anti-HEV IgG EIA reactivity in the general population of the Republic of Korea was estimated to be 5.9% between 2007 and 2009.

The anti-HEV IgG EIA reactivity of this study was the lowest compared to other studies in Korea, 11.9% in 2003 and 2004 [12], 9.41% in a previous KNHANES III study between 2004 and 2006 [11], and 14.3 ~ 23.1% in the group over the age of 20 years old in 2006 [16]. In addition, it was lower than that of Taiwan (12.9%) and [20] but was higher than Japan (5.3%), Italy (2.6%), Spain (2.17%), and Netherland (1.9%) [4, 21–23]. However, the seroprevalence data in these seroepidemiological studies should be compared with consideration of sensitivity and specificity of assays they used. For example, 28-50% of the anti-HEV EIA reactive samples were negative in the confirmatory test, recombinant immunoblot (RIB) [4, 23, 24]. In addition, among the anti-HEV EIA assay, Wantai assay has been reported to have greater sensitivity than Genelabs assay in previous studies [16, 25] and seropositivity of anti-HEV IgG was 14.3% in the Genelabs assay and 23.1% in the Wantai assay in Park *et al’*s study [16]. Therefore, we presented the seroprevalence data as frequency of anti-HEV reactivity rather than as anti-HEV prevalence, because we estimated anti-HEV seroprevalence using one assay, Wantai assay.

The present study demonstrated that older age, being male, low education level, working in agricultural, forestry, and fishery, and living in the costal and rural region were independently associated with risk of a past or recent exposure to HEV. The seroprevalence in men was higher than that in women. This was also observed by other studies, suggesting that men are at higher risk of developing disease following exposure compared to women [4, 26]. In addition, the strong association between age and HEV seroprevalence was revealed in our study and several previous studies [4, 11, 27–30], and it most likely reflects cumulative lifetime contact frequency to HEV. The analyses in this study showed that educational level was inversely related to the prevalence of anti-HEV antibodies. However, previous studies have indicated that there was no also no difference in anti-HEV seroprevalence according to educational status [31, 32]. The geographic variations in HEV seroprevalence in this study was appear to be influenced by the distribution of occupation in each region of Korea. The high seroprevalence regions had higher proportion of skilled agricultural, forestry, and fishery workers and elementary workers, Jeju-do (17.4%), Jeollabuk-do (17.1%), Jeollanam-do (17.9%), and Chungcheongnam-do (18.4%) than other regions such as Gyeonggi-do (4.9%) and Gyeongsangnam-do (6.1%) (data not shown).

Most cases of HEV infection in non-endemic countries have been associated with travel to HEV endemic regions, but sporadic autochthonous hepatitis E occur by zoonotic transmission [7, 12]. The results of this study supported the hypothesis that zoonotic transmission is an important risk factor for HEV infection in the republic of Korea. First, skilled agricultural, forestry, and fishery workers had the highest risk of being seropositive for HEV compared with other occupation group in our study. Similarly, professionals such as veterinarians and famers that are in frequent contact with animals, as well as people who frequently consume raw animal products, have a high prevalence of anti-HEV antibodies in other studies [9, 10, 12].

Second, previous epidemiological studies have identified consumption of HEV contaminated raw shellfish and oysters as an important risk factor for HEV infection [33, 34]. A recent study conducted in the republic of Korea showed that HEV genotype 3 that closely resembles a Korean strain of swine HEV was present in oysters and that the consumption of contaminated shellfish caused sporadic acute hepatitis E cases [15, 35]. The higher seroprevalence of coastal and rural areas in this study could be attributed to the more frequent contact with animals and raw shellfish. However, because the risk factors for HEV infection are not well defined, data for risk factor assessment could not be collected during the KNHANES IV study, resulting in our inability to conclusively examine the route of transmission or the extent of zoonotic HEV infections and the role of consumption of uncooked or undercooked meats HEV infections in the republic of Korea.

Overall, the seroprevalence of anti-HEV antibodies was evaluated by conducting a national survey and the association of serum anti-HEV antibodies with subject age, gender, region of residency, education level, and occupation was evaluated. Although HEV is non-endemic in the republic of Korea, the prevalence of anti-HEV antibodies in the Korean population was relatively higher than that of countries with non-endemic HEV. In addition, occupational exposure to infected animals and consuming contaminated food play a significant role in autochthonous hepatitis E in the republic of Korea, and several other identified potential associations, like older age and gender, consistent with findings in studies. Therefore, this study provides information that may be used for HEV risk factor assessment to prevent and reduce HEV infection in this country. Further studies that include detailed and continuous nationwide surveys are required to identify unrecognized risk factors and to monitor the HEV infection prevalence in the Republic of Korea.

## Authors’ original submitted files for images

Below are the links to the authors’ original submitted files for images. Authors’ original file for figure 1 Authors’ original file for figure 2 Authors’ original file for figure 3
