# Supplementary figures and images for: Hepatitis E Virus (HEV) Seroprevalence in the general population of the Republic of Korea in 2007–2009: a nationwide cross-sectional study
Source: BMC Infect Dis. 2014 Sep 24;14:517. doi: 10.1186/1471-2334-14-517 (PMC4262127; doi:10.1186/1471-2334-14-517)

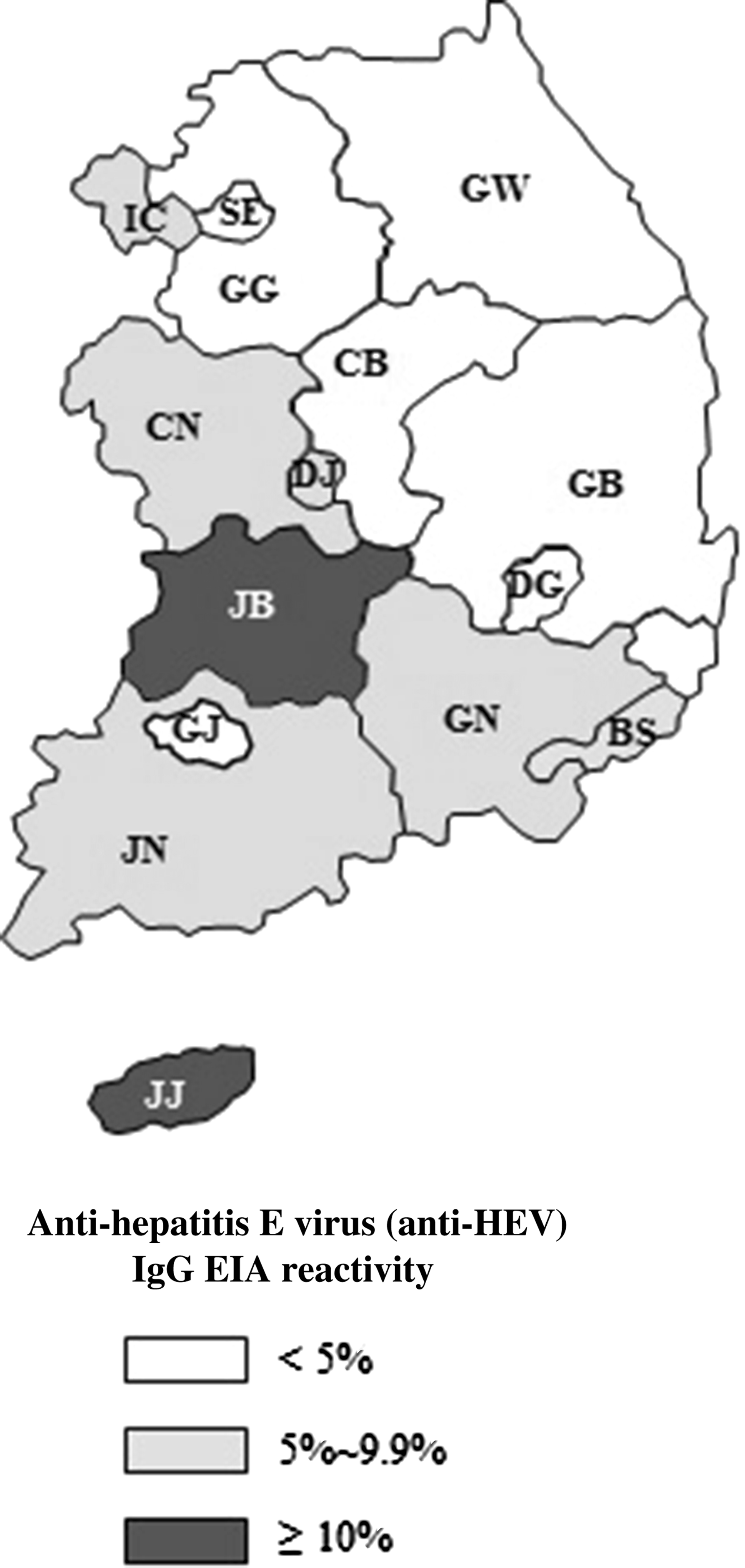

Supplement: Supplementary file 1 — Authors’ original file for figure 1 [file 12879_2014_3853_MOESM1_ESM.tiff]

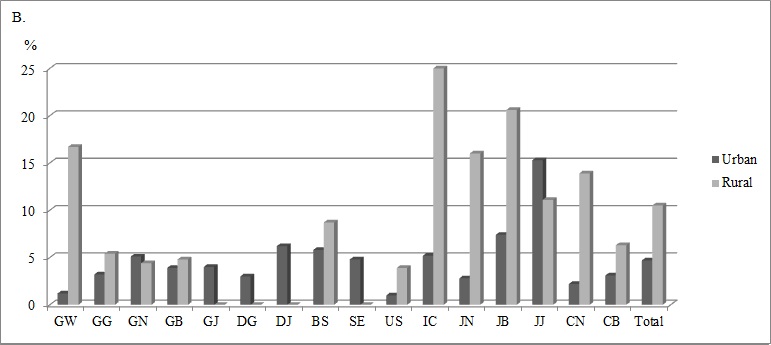

Supplement: Supplementary file 2 — Authors’ original file for figure 2 [file 12879_2014_3853_MOESM2_ESM.jpeg]

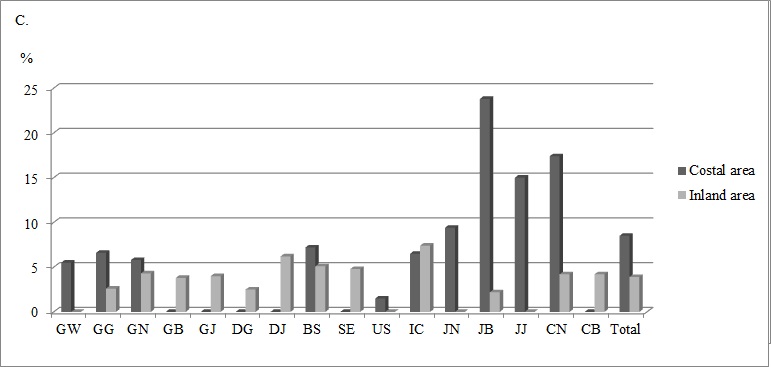

Supplement: Supplementary file 3 — Authors’ original file for figure 3 [file 12879_2014_3853_MOESM3_ESM.jpeg]
